# Paravertebral block reduces pain in elderly patients with percutaneous nephrolithotomy: A randomized controlled study protocol

**DOI:** 10.1097/MD.0000000000023761

**Published:** 2020-12-18

**Authors:** Jun Deng, Ke Wei, Mingliang Li, Xiaoping Wang, Qianli Tang

**Affiliations:** a Graduation School, Jinan University, Guangzhou; b Department of Anesthesiology, Affiliated Hospital of Youjiang Medical University for Nationalities, Guangxi; c Department of Pain Management, the First Hospital of Jinan University, Guangzhou; d Department of Gastrointestinal surgery, Affiliated Hospital of Youjiang Medical University for Nationalities, Guangxi, China.

**Keywords:** percutaneous nephrolithotomy, paravertebral block, pain, protocol

## Abstract

**Objective:**

To assess the effectiveness of paravertebral block for the percutaneous nephrolithotomy (PCNL) patients.

**Method:**

This study will be implemented from May 2021 to March 2022 at Affiliated Hospital of Youjiang Medical University for Nationalities and it was granted through the Research Ethics Committee of Affiliated Hospital of Youjiang Medical University for Nationalities (No.60192038). This study includes a total of 100 patients. The criteria for inclusion of patients involves:

1. patients over 60 years old;
2. American Society of Anaesthesiologists I or II classification.

The criteria for exclusion involves:

1. skin infection at injection site, spinal deformity, abnormal coagulation function;
2. patient refusal, and known drug allergy.

The visual analogue scores, heart rate, the diastolic and systolic blood pressure, complications, and side effects, the consumption of opioid and extra analgesic needs are recorded in rehabilitation room 1 hour after the surgery and in the first 24 hours of urological service.

**Results:**

Table 1 shows the postoperative data among 2 groups.

**Conclusion:**

In comparison with traditional analgesia, the ultrasound-guided paraventric block is an effective analgesic approach in PCNL, and no additional complications are encountered.

**Trial registration number:**

research registry 6259.

## 1 Introduction

Stone disease is a very familiar disorder, uniting all the countries in the world, despite the surgical treatment will be determined by many factors, containing the availability of distinct technologies.^[1,2]^ For urolithiasis, its epidemiology varies on the basis of the prevalence and incidence rate, sex and age distribution, location of stones and composition of stones.^[3,4]^ These differences can be explained by climate, diet and race, among other factors. A review of former epidemiological surveys indicated that the prevalence rate is between 4 percent and 20 percent in the economically developed countries.^[5]^

Percutaneous nephrolithotomy (PCNL) is a kind of minimally invasive procedure, which has been extensively utilized in the treatment of kidney stones.^[6,7]^ It is still a gold standard for treating the kidney stones due to it has less morbidity and invasion than open surgery. Nevertheless, PCNL can lead to the pain after operation and pelvicalyceal system distension owing to the inserted nephrostomy. Many approaches, such as epidural analgesics, non-steroidal anti-inflammatory drugs, and systemic opioids, have been developed to relieve pain, decrease the related complications, hospitalization and medical costs.^[8,9]^ Nevertheless, these approaches may lead to serious adverse reactions, for instance, nausea and vomiting, sedation, respiratory depression, kidney problems, and constipation, particularly in the elderly patients.^[10]^ In a variety of surgeries, paravertebral block is a successful approach of local analgesia.^[11]^ It can generate sympathetic, somatosensory and unilateral block by injecting local anesthetics into paravertebral space containing the thoracic spinal nerves and its branches. The former studies have demonstrated its analgesic effect in abdominal surgery, breast surgery, and thoracotomy.^[12–14]^ At present, there is insufficient evidence for the conventional clinical application of paravertebral block in the PCNL, and some investigations have explored the safety and effectiveness of the paravertebral block in these patients. Therefore, we perform this randomized controlled study protocol to assess the effectiveness of paravertebral block for the PCNL patients.

## 2 Methods

This study will be implemented from May 2021 to March 2022 at Affiliated Hospital of Youjiang Medical University for Nationalities. The experiment was granted through the Research Ethics Committee of Affiliated Hospital of Youjiang Medical University for Nationalities (No. 60192038) and recorded in research registry (researchregistry6259). Sequence of random numbers is generated by a computer. Sequentially numbered sealed opaque envelopes are used for the concealment of random numbers. All the patients taking part in our experiment are randomly assigned into the study group and the control group, and each group includes 50 patients.

### 2.1 Inclusion and exclusion criteria

This study includes a total of 100 patients. The criteria for inclusion of patients involves:

1. patients over 60 years old;
2. American Society of Anaesthesiologists I or II classification.

The criteria for exclusion involves:

1. skin infection at injection site, spinal deformity, abnormal coagulation function;
2. patient refusal, and known drug allergy.

### 2.2 Paravertebral block

The patients do not receive any premedication. All the patients are given standard monitoring in operating room, involving the saturation of peripheral blood and noninvasive arterial blood pressure. After intravenous anesthesia with 2 μg/kg of fentanyl, 0.5 mg/kg of rocuronium bromide, and 2 mg/kg of propofol, all the patient are intubated by using the appropriate endotracheal tube. Anesthesia is maintained with a gaseous mixture of 40% oxygen and 60% nitrous oxide, and 1% to 2% sevoflurane, and the controlled ventilation is utilized. 0.9% NaCl (5–10 ml/kg) is utilized for the fluid resuscitation. After the urinary catheter is inserted, patients are placed in a prone position. Prior to surgery, under the guidance of ultrasound, the paravertebral block is administered with 0.5% of bupivacaine at L1, T12, and T11 levels, and a total dose of bupivacaine is 15 ml. The injection site is cleaned by using a 10% solution of povidone iodine and then covers via the sterile drapes. Meanwhile, under the sterile conditions, the linear detection probe can be prepared. On ultrasound, the paravertovertebral space refers to the area between the transverse process, the pleura, and costotransverse ligament. Through utilizing the in-plane technology, a 22-gauge insulated echogenic needle is advanced in the vertical-to-caudal direction. After inserting the needle into paracervical space, 0.5% of bupivacaine (5 ml) is injected subcutaneously in each segment, which is controlled via aspiration. The diffusion of local anesthetics can be demonstrated via the forward movement of pleura in paravertebral space. Control group receives intravenous tramadol for pain control.

### 2.3 Outcomes

The visual analogue score, heart rate, the diastolic and systolic blood pressure, complications, side effects, the consumption of opioid and extra analgesic needs are recorded in rehabilitation room 1 hour after the surgery and in the first 24 hours of urological service. At rest, the pain scores are assessed on the basis of visual analogue scale for pain (in which 0 cm represents no pain; while 10 cm represents the most severe pain).

### 2.4 Statistical analysis

Through utilizing the Microsoft Excel 2013, the data is recorded, and the analysis of all the data is carried out through utilizing the software of IBM SPSS Statistics for Windows, version 20 (IBM Corp., Armonk, NY, USA). All the data are represented via the proper characteristics, for instance, median, mean and percentage. The categorical variables and continuous variables are respectively analyzed using independent *t* tests and χ^2^-tests. *P* value less than .05 indicates that there is statistical significance.

## 3 Results

Table 1 shows the postoperative data among 2 groups.

**Table 1:** The postoperative data among 2 groups.

Variables	Paraventric block group (n = 50)	Control group (n = 50)	*P* value
Postoperative pain score at 4 hours			
Postoperative pain score at 12 hours			
Postoperative pain score at 24 hours			
Total opioid consumption			
Postoperative complications			
Length of stay			

## 4 Discussion

PCNL plays a prominent role in treating the urinary system stone diseases.^[15,16]^ Postoperative pain often appears after receiving PCNL, which results in prolonged hospitalization, decreased life quality, and then develops into chronic pain, accompanied by pulmonary and thromboembolic complications, circulatory failure, respiratory depression, and other complications, such as urinary retention, intestinal dysfunction, sleep disorders, nausea and vomiting. Peripheral nerve block, infiltration of local anesthesia and intravenous opioids are the analgesic techniques utilized for the control of postoperative pain in the adult patients receiving PCNL. Nevertheless, the optimal approaches is still controversial. Paravertebral block has been demonstrated to be effective in controlling postoperative pain in patients undergoing a variety of operations, and has no significant effect on hemodynamic balance, bowel movements or motor blockade.^[17]^ The ultrason-guided paravertebral block can display the target nerve, needle as well as the adjacent anatomical structures in real time, and the application of local anesthesia can not only reduce the incidence of complications and failure rate, but also reduce the dose of local anesthesia.^[18]^ Due to the sample number of included patients, future study is required.

## 5 Conclusion

In comparison with traditional analgesia, the ultrasound-guided paraventric block is an effective analgesic approach in PCNL, and no additional complications are encountered.

## Author contributions

Qianli Tang and Xiaoping Wang plan the study design. Mingliang Li reviews the protocol. Ke Wei will collect data. Jun Deng writes the manuscript. All authors approve the submission.

**Conceptualization:** Ke Wei.

**Funding acquisition:** Qianli Tang.

**Investigation:** Mingliang Li.

**Project administration:** Xiaoping Wang.

**Writing – original draft:** Jun Deng.

## References

[1] Sorokin I Mamoulakis C Miyazawa K Epidemiology of stone disease across the world. World J Urol 2017;35:1301–20. 28213860 10.1007/s00345-017-2008-6

[2] Canvasser NE Alken P Lipkin M The economics of stone disease. World J Urol 2017;35:1321–9. 28108799 10.1007/s00345-017-2003-y

[3] Abou-Elela A Epidemiology, pathophysiology, and management of uric acid urolithiasis: a narrative review. J Adv Res 2017;8:513–27. 28748117 10.1016/j.jare.2017.04.005 PMC5512151

[4] Trinchieri A Epidemiology of urolithiasis: an update. Clin Cases Miner Bone Metab 2008;5:101–6. 22460989 PMC2781200

[5] Bartoletti R Cai T Mondaini N Epidemiology and risk factors in urolithiasis. Urol Int 2007;79: Suppl 1: 3–7. 17726345 10.1159/000104434

[6] Jones P Aboumarzouk OM Rai BP Percutaneous nephrolithotomy for stones in solitary kidney: evidence from a systematic review. Urology 2017;103:12–8. 27856206 10.1016/j.urology.2016.10.022

[7] Guler A Erbin A Ucpinar B Comparison of miniaturized percutaneous nephrolithotomy and standard percutaneous nephrolithotomy for the treatment of large kidney stones: a randomized prospective study. Urolithiasis 2019;47:289–95. 29858913 10.1007/s00240-018-1061-y

[8] Dundar G Gokcen K Gokce G The effect of local anesthetic agent infiltration around nephrostomy tract on postoperative pain control after percutaneous nephrolithotomy: a single-centre, randomised, double-blind, placebocontrolled clinical trial. Urol J 2018;15:306–12. 29681047 10.22037/uj.v0i0.4145

[9] Arshad Z Zaidi SZ Jamshaid A Post operative pain control in percutaneous nephrolithotomy. J Pak Med Assoc 2018;68:702–4. 29885165

[10] Kane-Gill SL Rubin EC Smithburger PL The cost of opioid-related adverse drug events. J Pain Palliat Care Pharmacother 2014;28:282–93. 25102043 10.3109/15360288.2014.938889

[11] Baldea KG Patel PM Delos SG Paravertebral block for percutaneous nephrolithotomy: a prospective, randomized, double-blind placebo-controlled study. World J Urol 2020;38:2963–9. 31982963 10.1007/s00345-020-03093-3

[12] Heesen M Klimek M Rossaint R Paravertebral block and persistent postoperative pain after breast surgery: meta-analysis and trial sequential analysis. Anaesthesia 2016;71:1471–81. 27714754 10.1111/anae.13649

[13] Yeung JH Gates S Naidu BV Paravertebral block versus thoracic epidural for patients undergoing thoracotomy. Cochrane Database Syst Rev 2016;2:D9121. 10.1002/14651858.CD009121.pub2 PMC7151756 26897642

[14] Page EA Taylor KL Paravertebral block in paediatric abdominal surgery-a systematic review and meta-analysis of randomized trials. Br J Anaesth 2017;118:159–66. 28100519 10.1093/bja/aew387

[15] Agrawal MS Agarwal K Jindal T Ultra-mini-percutaneous nephrolithotomy: a minimally-invasive option for percutaneous stone removal. Indian J Urol 2016;32:132–6. 27127356 10.4103/0970-1591.174778 PMC4831502

[16] Rizvi S Hussain M Askari SH Surgical outcomes of percutaneous nephrolithotomy in 3402 patients and results of stone analysis in 1559 patients. BJU INT 2017;120:702–9. 28303631 10.1111/bju.13848

[17] Shimizu H Kamiya Y Nishimaki H Thoracic paravertebral block reduced the incidence of chronic postoperative pain for more than 1 year after breast cancer surgery. JA Clin Rep 2015;1:19. 29497651 10.1186/s40981-015-0023-4 PMC5818708

[18] Zeballos JL Voscopoulos C Kapottos M Ultrasound-guided retrolaminar paravertebral block. Anaesthesia 2013;68:649–51. 10.1111/anae.12296 23662765

